# *p*-Tolyl­methanaminium cyclo­hexane-1,2-diyl phosphate

**DOI:** 10.1107/S1600536810044326

**Published:** 2010-11-06

**Authors:** Ravikumar R. Gowda, Venkatachalam Ramkumar, Debashis Chakraborty

**Affiliations:** a Department of Chemistry, IIT Madras, Chennai, TamilNadu, India

## Abstract

In the title mol­ecular salt, C_8_H_12_N^+^·C_6_H_10_O_4_P^−^, the cation and anion are connected by N—H⋯O hydrogen bonds. The C atoms of the cyclo­hexane ring are disordered over two sets of sites in a 0.51 (4):0.49 (4) occupancy ratio to generate two superimposed chair conformations. One of the terminal phosphate O atoms is also disordered in a 0.62 (2):0.38 (2) ratio.

## Related literature

For a related structure and background to organic phosphates, see: Gowda *et al.* (2010 ▶). For ring-puckering parameters, see: Cremer & Pople (1975 ▶).
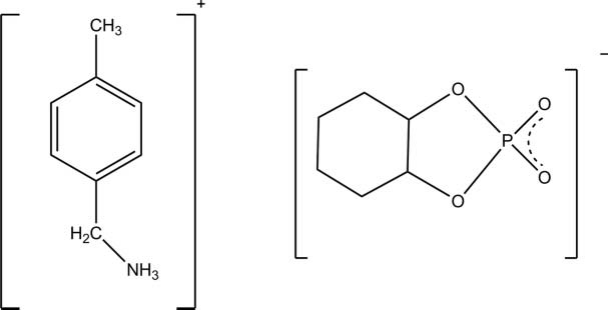


## Experimental

### 

#### Crystal data

C_8_H_12_N^+^·C_6_H_10_O_4_P^−^
*M* *_r_* = 299.30
Triclinic, 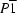

*a* = 5.9642 (6) Å
*b* = 9.6077 (10) Å
*c* = 13.7070 (15) Å
α = 78.326 (6)°
β = 82.549 (7)°
γ = 84.900 (6)°
*V* = 761.11 (14) Å^3^
*Z* = 2
Mo *K*α radiation
μ = 0.19 mm^−1^
*T* = 298 K
0.22 × 0.20 × 0.15 mm

#### Data collection

Bruker APEXII CCD diffractometer
Absorption correction: multi-scan (*SADABS*; Bruker, 1999 ▶) *T*_min_ = 0.959, *T*_max_ = 0.972
9372 measured reflections
3112 independent reflections
1549 reflections with *I* > 2σ(*I*)
*R*_int_ = 0.070

#### Refinement

*R*[*F* ^2^ > 2σ(*F* ^2^)] = 0.068
*wR*(*F* ^2^) = 0.177
*S* = 0.99
3112 reflections
217 parameters
H atoms treated by a mixture of independent and constrained refinement
Δρ_max_ = 0.29 e Å^−3^
Δρ_min_ = −0.26 e Å^−3^

### 

Data collection: *APEX2* (Bruker, 2004 ▶); cell refinement: *APEX2* and *SAINT* (Bruker, 2004 ▶); data reduction: *SAINT* and *XPREP* (Bruker, 2004 ▶); program(s) used to solve structure: *SHELXS97* (Sheldrick, 2008 ▶); program(s) used to refine structure: *SHELXL97* (Sheldrick, 2008 ▶); molecular graphics: *ORTEP-3* (Farrugia, 1997 ▶); software used to prepare material for publication: *SHELXL97*.

## Supplementary Material

Crystal structure: contains datablocks global, I. DOI: 10.1107/S1600536810044326/hb5717sup1.cif

Structure factors: contains datablocks I. DOI: 10.1107/S1600536810044326/hb5717Isup2.hkl

Additional supplementary materials:  crystallographic information; 3D view; checkCIF report

## Figures and Tables

**Table 1:** Hydrogen-bond geometry (Å, °)

*D*—H⋯*A*	*D*—H	H⋯*A*	*D*⋯*A*	*D*—H⋯*A*
N1—H3N⋯O3^i^	0.92 (4)	1.90 (4)	2.788 (5)	161 (3)
N1—H2N⋯O3^ii^	1.00 (6)	1.86 (6)	2.834 (5)	164 (4)
N1—H1N⋯O4^iii^	0.92 (5)	1.72 (6)	2.63 (2)	171 (5)

Symmetry codes: (i) 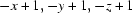; (ii) 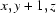; (iii) 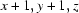.

**Fig. 1.**
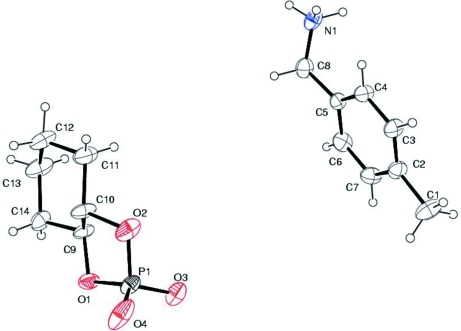
View of (I) with atoms represented as 30% probability ellipsoids.

**Fig. 2.**
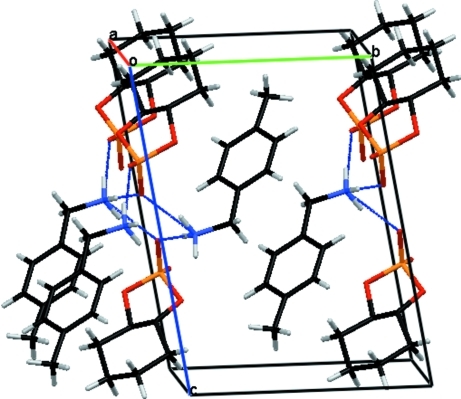
The packing diagram showing the N—H···O interactions.

## supplementary crystallographic information

### Comment

As part of our ongoing studies of organic phosphates (Gowda *et al.*, 2010), we now report the structure of the title salt (I) of cyclohexanediol phosphoric acid instead of binol phosphoric acid as compared to earlier report.

The cyclohexane ring is puckered and the ring puckering parameters such as total puckering amplitude Q_T_ and phase angle θ are 0.615 (14) Å and 8.3 (15)° respectively, the q_2_ and q_3_ are 0.076 (16) and 0.609 (15) Å, respectively (Cremer & Pople, 1975). Thus, all parameters strongly support the near ideal chair conformation for the cyclohexane ring C9–C14.

The C atoms of the cyclohexane ring are disordered, with a site-occupancy factor of 0.51 (4) for the major component and the O atom attached to the phosphate is also disordered, with a site-occupancy factor of 0.62 (2).

The N atom in the *p*-tolyl methanammonium cation exhibits a trigonal pyramidal coordinate geometry with three phosphate O atom forming three N—H···O interactions.

### Experimental

To an stirred ice cold solution of 0.15 g (1.29 mmol) *trans*-1,2-cyclohexanediol in 10 ml of dichloromethane under nitrogen atmosphere was added 0.12 ml (1.29 mmol) POCl_3_ drop wise followed by addition of 3.6 ml (25.8 mmol) triethylamine. White fumes of HCl were observed upon addition, reaction mixture was stirred at 273 K for 30 min. Then 0.8 ml (6.5 mmol) 4-methylbenzylamine was added slowly at 273 K. Reaction mixture was stirred at 273 K for 1 h and warmed up to room temperature and stirred for 48 h. The reaction was monitored using thin layer chromatography. The reaction mixture was then washed with 2 ml of water. The product was extracted using dichloromethane and purified by crystallization in dichloromethane to yield colourless blocks of (I).

### Refinement

All H atoms except the nitrogen H atoms were fixed geometrically and allowed to ride on the parent C atoms with aromatic C—H = 0.93 Å, aliphatic C—H = 0.98 Å, methine C—H = 0.97 Å, methylene C—H = 0.97 Å and methyl C—H = 0.96 Å. The displacement parameters were set for phenyl, methine and aliphatic H atoms at *U*_iso_(H) = 1.2*U*_eq_(C) and methyl H atoms at *U*_iso_(H) = 1.5*U*_eq_(C)

The cyclohexane ring  C9–C14 are disordered in two orientations with refined site occupancy of 0.51 (4) and 0.49 (5) respectively. The O atom attached to the phosphate is also disordered, with a site-occupancy factor of 0.62 (2) and 0.38 (3) respectively. Some anisotropic displacement ellipsoids were rather elongated which led us to use the EADP restraints.

### Crystal data

**Table:**

C_8_H_12_N^+^·C_6_H_10_O_4_P^−^	*Z* = 2
*M**_r_* = 299.30	*F*(000) = 320
Triclinic, *P*1	*D*_x_ = 1.306 Mg m^−^^3^
Hall symbol: -P 1	Mo *K*α radiation, λ = 0.71073 Å
*a* = 5.9642 (6) Å	Cell parameters from 1420 reflections
*b* = 9.6077 (10) Å	θ = 2.4–19.6°
*c* = 13.7070 (15) Å	µ = 0.19 mm^−^^1^
α = 78.326 (6)°	*T* = 298 K
β = 82.549 (7)°	Block, colourless
γ = 84.900 (6)°	0.22 × 0.20 × 0.15 mm
*V* = 761.11 (14) Å^3^	

### Data collection

**Table:**

Bruker APEXII CCD diffractometer	3112 independent reflections
Radiation source: fine-focus sealed tube	1549 reflections with *I* > 2σ(*I*)
graphite	*R*_int_ = 0.070
φ and ω scans	θ_max_ = 27.4°, θ_min_ = 2.2°
Absorption correction: multi-scan (*SADABS*; Bruker, 1999)	*h* = −7→6
*T*_min_ = 0.959, *T*_max_ = 0.972	*k* = −12→12
9372 measured reflections	*l* = −17→17

### Refinement

**Table:**

Refinement on *F*^2^	Primary atom site location: structure-invariant direct methods
Least-squares matrix: full	Secondary atom site location: difference Fourier map
*R*[*F*^2^ > 2σ(*F*^2^)] = 0.068	Hydrogen site location: inferred from neighbouring sites
*wR*(*F*^2^) = 0.177	H atoms treated by a mixture of independent and constrained refinement
*S* = 0.99	*w* = 1/[σ^2^(*F*_o_^2^) + (0.0845*P*)^2^] where *P* = (*F*_o_^2^ + 2*F*_c_^2^)/3
3112 reflections	(Δ/σ)_max_ = 0.002
217 parameters	Δρ_max_ = 0.28 e Å^−^^3^
0 restraints	Δρ_min_ = −0.25 e Å^−^^3^

### Special details

**Table:**

Geometry. All esds (except the esd in the dihedral angle between two l.s. planes) are estimated using the full covariance matrix. The cell esds are taken into account individually in the estimation of esds in distances, angles and torsion angles; correlations between esds in cell parameters are only used when they are defined by crystal symmetry. An approximate (isotropic) treatment of cell esds is used for estimating esds involving l.s. planes.
Refinement. Refinement of F^2^ against ALL reflections. The weighted R-factor wR and goodness of fit S are based on F^2^, conventional R-factors R are based on F, with F set to zero for negative F^2^. The threshold expression of F^2^ > 2sigma(F^2^) is used only for calculating R-factors(gt) etc. and is not relevant to the choice of reflections for refinement. R-factors based on F^2^ are statistically about twice as large as those based on F, and R- factors based on ALL data will be even larger.

### Fractional atomic coordinates and isotropic or equivalent isotropic displacement parameters (Å^2^)

**Table:**

	*x*	*y*	*z*	*U*_iso_*/*U*_eq_	Occ. (<1)
C1	0.1252 (8)	0.4020 (5)	0.8217 (3)	0.0796 (14)	
H1A	−0.0022	0.4646	0.8385	0.119*	
H1B	0.2040	0.3678	0.8796	0.119*	
H1C	0.0731	0.3228	0.7999	0.119*	
C2	0.2837 (6)	0.4820 (4)	0.7383 (3)	0.0494 (10)	
C3	0.2204 (6)	0.6086 (4)	0.6806 (3)	0.0473 (10)	
H3	0.0751	0.6492	0.6943	0.057*	
C4	0.3634 (6)	0.6791 (4)	0.6024 (3)	0.0456 (10)	
H4	0.3141	0.7655	0.5647	0.055*	
C5	0.5811 (6)	0.6207 (4)	0.5803 (3)	0.0388 (9)	
C6	0.6472 (6)	0.4933 (4)	0.6386 (3)	0.0533 (11)	
H6	0.7928	0.4529	0.6257	0.064*	
C7	0.5009 (7)	0.4247 (4)	0.7160 (3)	0.0567 (11)	
H7	0.5492	0.3382	0.7539	0.068*	
C8	0.7453 (6)	0.6848 (4)	0.4931 (3)	0.0519 (10)	
H8A	0.8979	0.6640	0.5118	0.062*	
H8B	0.7349	0.6389	0.4371	0.062*	
C9	0.5555 (16)	0.0572 (10)	0.1899 (7)	0.0359 (17)	0.511 (5)
H9	0.6268	0.0962	0.2381	0.043*	0.511 (5)
C10	0.3502 (14)	0.1493 (9)	0.1592 (7)	0.0414 (17)	0.511 (5)
H10	0.2784	0.1120	0.1103	0.050*	0.511 (5)
C12	0.595 (3)	0.298 (2)	0.0267 (13)	0.071 (4)	0.511 (5)
H12A	0.6495	0.3920	0.0012	0.085*	0.511 (5)
H12B	0.5233	0.2705	−0.0255	0.085*	0.511 (5)
C13	0.802 (3)	0.189 (3)	0.0537 (14)	0.071 (4)	0.511 (5)
H13A	0.9034	0.1825	−0.0068	0.085*	0.511 (5)
H13B	0.8849	0.2224	0.0997	0.085*	0.511 (5)
C9A	0.4735 (15)	0.0466 (11)	0.1512 (7)	0.0359 (17)	0.489 (5)
H9A	0.3655	0.0470	0.1030	0.043*	0.489 (5)
C10A	0.4392 (14)	0.1751 (10)	0.1959 (7)	0.0414 (17)	0.489 (5)
H10A	0.5531	0.1797	0.2405	0.050*	0.489 (5)
C12A	0.683 (3)	0.302 (2)	0.0589 (14)	0.071 (4)	0.489 (5)
H12C	0.6978	0.3842	0.0048	0.085*	0.489 (5)
H12D	0.7896	0.3070	0.1057	0.085*	0.489 (5)
C13A	0.736 (3)	0.168 (3)	0.0176 (15)	0.071 (4)	0.489 (5)
H13C	0.8870	0.1693	−0.0183	0.085*	0.489 (5)
H13D	0.6291	0.1636	−0.0292	0.085*	0.489 (5)
C11	0.4260 (7)	0.3024 (4)	0.1158 (3)	0.0682 (13)	
H11A	0.2961	0.3656	0.0968	0.082*	
H11B	0.4924	0.3382	0.1660	0.082*	
C14	0.7203 (6)	0.0391 (4)	0.1023 (3)	0.0541 (11)	
H14A	0.6492	0.0002	0.0549	0.065*	
H14B	0.8474	−0.0252	0.1234	0.065*	
N1	0.7087 (6)	0.8402 (4)	0.4595 (3)	0.0508 (9)	
O1	0.4388 (4)	−0.0703 (2)	0.23999 (19)	0.0548 (8)	
O2	0.2054 (4)	0.1419 (3)	0.2529 (2)	0.0667 (9)	
O3	0.2813 (4)	−0.0294 (3)	0.41168 (19)	0.0606 (8)	
O4	0.001 (9)	−0.072 (10)	0.3026 (15)	0.089 (12)	0.62 (14)
O4A	0.051 (6)	−0.119 (3)	0.299 (2)	0.054 (6)	0.38 (14)
P1	0.22204 (15)	−0.01973 (11)	0.30949 (8)	0.0446 (4)	
H3N	0.720 (6)	0.884 (4)	0.512 (3)	0.048 (12)*	
H2N	0.552 (10)	0.869 (5)	0.440 (4)	0.12 (2)*	
H1N	0.816 (9)	0.861 (5)	0.405 (4)	0.089 (16)*	

### Atomic displacement parameters (Å^2^)

**Table:**

	*U*^11^	*U*^22^	*U*^33^	*U*^12^	*U*^13^	*U*^23^
C1	0.067 (3)	0.085 (3)	0.073 (3)	−0.026 (2)	0.009 (3)	0.015 (3)
C2	0.044 (2)	0.053 (2)	0.050 (2)	−0.0162 (18)	0.0016 (19)	−0.007 (2)
C3	0.031 (2)	0.054 (2)	0.053 (2)	−0.0035 (16)	0.0041 (18)	−0.005 (2)
C4	0.037 (2)	0.042 (2)	0.053 (2)	−0.0010 (16)	0.0000 (18)	−0.0012 (18)
C5	0.0311 (19)	0.043 (2)	0.043 (2)	−0.0065 (15)	0.0013 (17)	−0.0117 (18)
C6	0.037 (2)	0.055 (3)	0.068 (3)	0.0015 (18)	0.000 (2)	−0.015 (2)
C7	0.060 (3)	0.041 (2)	0.066 (3)	0.0009 (19)	−0.009 (2)	−0.003 (2)
C8	0.042 (2)	0.062 (3)	0.051 (3)	−0.0062 (18)	0.0028 (19)	−0.013 (2)
C9	0.032 (5)	0.039 (3)	0.033 (6)	−0.005 (4)	−0.002 (3)	0.001 (4)
C10	0.024 (4)	0.045 (4)	0.049 (5)	−0.005 (3)	−0.002 (3)	0.005 (3)
C12	0.068 (12)	0.067 (4)	0.060 (11)	−0.009 (8)	0.005 (6)	0.022 (7)
C13	0.037 (9)	0.082 (8)	0.076 (12)	−0.013 (6)	0.013 (6)	0.017 (9)
C9A	0.032 (5)	0.039 (3)	0.033 (6)	−0.005 (4)	−0.002 (3)	0.001 (4)
C10A	0.024 (4)	0.045 (4)	0.049 (5)	−0.005 (3)	−0.002 (3)	0.005 (3)
C12A	0.068 (12)	0.067 (4)	0.060 (11)	−0.009 (8)	0.005 (6)	0.022 (7)
C13A	0.037 (9)	0.082 (8)	0.076 (12)	−0.013 (6)	0.013 (6)	0.017 (9)
C11	0.072 (3)	0.046 (2)	0.071 (3)	0.004 (2)	0.012 (2)	0.009 (2)
C14	0.043 (2)	0.063 (3)	0.047 (2)	0.0031 (18)	0.0098 (19)	−0.002 (2)
N1	0.036 (2)	0.074 (3)	0.041 (2)	−0.0198 (17)	0.0038 (18)	−0.005 (2)
O1	0.0565 (17)	0.0393 (15)	0.0598 (17)	−0.0088 (12)	0.0209 (13)	−0.0036 (13)
O2	0.0441 (17)	0.0680 (18)	0.0662 (19)	0.0195 (13)	0.0250 (14)	0.0087 (15)
O3	0.0517 (18)	0.086 (2)	0.0418 (17)	−0.0123 (14)	−0.0052 (13)	−0.0030 (14)
O4	0.047 (11)	0.17 (3)	0.047 (6)	−0.058 (16)	−0.005 (5)	0.007 (8)
O4A	0.028 (8)	0.068 (17)	0.071 (11)	−0.025 (6)	0.007 (6)	−0.023 (9)
P1	0.0272 (5)	0.0603 (7)	0.0411 (7)	−0.0103 (4)	0.0019 (4)	0.0019 (5)

### Geometric parameters (Å, °)

**Table:**

C1—C2	1.511 (5)	C13—C14	1.55 (2)
C1—H1A	0.9600	C13—H13A	0.9700
C1—H1B	0.9600	C13—H13B	0.9700
C1—H1C	0.9600	C9A—C10A	1.474 (17)
C2—C3	1.362 (5)	C9A—O1	1.486 (10)
C2—C7	1.383 (5)	C9A—C14	1.538 (10)
C3—C4	1.381 (5)	C9A—H9A	0.9800
C3—H3	0.9300	C10A—C11	1.473 (10)
C4—C5	1.390 (5)	C10A—O2	1.534 (8)
C4—H4	0.9300	C10A—H10A	0.9800
C5—C6	1.376 (5)	C12A—C13A	1.50 (4)
C5—C8	1.507 (5)	C12A—C11	1.628 (19)
C6—C7	1.378 (5)	C12A—H12C	0.9700
C6—H6	0.9300	C12A—H12D	0.9700
C7—H7	0.9300	C13A—C14	1.52 (2)
C8—N1	1.476 (5)	C13A—H13C	0.9700
C8—H8A	0.9700	C13A—H13D	0.9700
C8—H8B	0.9700	C11—H11A	0.9700
C9—O1	1.464 (9)	C11—H11B	0.9700
C9—C14	1.478 (9)	C14—H14A	0.9700
C9—C10	1.500 (16)	C14—H14B	0.9700
C9—H9	0.9800	N1—H3N	0.92 (4)
C10—O2	1.445 (8)	N1—H2N	1.00 (6)
C10—C11	1.555 (9)	N1—H1N	0.92 (5)
C10—H10	0.9800	O1—P1	1.603 (2)
C12—C11	1.48 (2)	O2—P1	1.590 (3)
C12—C13	1.58 (3)	O3—P1	1.471 (3)
C12—H12A	0.9700	O4—P1	1.471 (17)
C12—H12B	0.9700	O4A—P1	1.50 (3)
			
C2—C1—H1A	109.5	O2—C10A—H10A	112.8
C2—C1—H1B	109.5	C13A—C12A—C11	109.5 (12)
H1A—C1—H1B	109.5	C13A—C12A—H12C	109.8
C2—C1—H1C	109.5	C11—C12A—H12C	109.8
H1A—C1—H1C	109.5	C13A—C12A—H12D	109.8
H1B—C1—H1C	109.5	C11—C12A—H12D	109.8
C3—C2—C7	117.3 (3)	H12C—C12A—H12D	108.2
C3—C2—C1	122.7 (4)	C12A—C13A—C14	110.0 (14)
C7—C2—C1	119.9 (4)	C12A—C13A—H13C	109.7
C2—C3—C4	122.6 (3)	C14—C13A—H13C	109.7
C2—C3—H3	118.7	C12A—C13A—H13D	109.7
C4—C3—H3	118.7	C14—C13A—H13D	109.7
C3—C4—C5	119.8 (3)	H13C—C13A—H13D	108.2
C3—C4—H4	120.1	C10A—C11—C12	114.1 (9)
C5—C4—H4	120.1	C10A—C11—C10	32.9 (4)
C6—C5—C4	118.0 (3)	C12—C11—C10	108.7 (9)
C6—C5—C8	118.0 (3)	C10A—C11—C12A	101.9 (8)
C4—C5—C8	123.9 (3)	C12—C11—C12A	26.9 (7)
C5—C6—C7	121.0 (4)	C10—C11—C12A	112.1 (9)
C5—C6—H6	119.5	C10A—C11—H11A	129.8
C7—C6—H6	119.5	C12—C11—H11A	109.9
C6—C7—C2	121.3 (4)	C10—C11—H11A	109.9
C6—C7—H7	119.4	C12A—C11—H11A	128.1
C2—C7—H7	119.4	C10A—C11—H11B	78.0
N1—C8—C5	114.7 (3)	C12—C11—H11B	109.9
N1—C8—H8A	108.6	C10—C11—H11B	109.9
C5—C8—H8A	108.6	C12A—C11—H11B	84.3
N1—C8—H8B	108.6	H11A—C11—H11B	108.3
C5—C8—H8B	108.6	C9—C14—C13A	115.1 (10)
H8A—C8—H8B	107.6	C9—C14—C9A	30.4 (3)
O1—C9—C14	115.2 (7)	C13A—C14—C9A	105.0 (9)
O1—C9—C10	97.5 (6)	C9—C14—C13	106.8 (9)
C14—C9—C10	111.7 (7)	C13A—C14—C13	27.7 (7)
O1—C9—H9	110.6	C9A—C14—C13	111.9 (9)
C14—C9—H9	110.6	C9—C14—H14A	110.4
C10—C9—H9	110.6	C13A—C14—H14A	82.9
O2—C10—C9	102.6 (7)	C9A—C14—H14A	80.9
O2—C10—C11	112.0 (6)	C13—C14—H14A	110.4
C9—C10—C11	107.8 (6)	C9—C14—H14B	110.4
O2—C10—H10	111.4	C13A—C14—H14B	125.1
C9—C10—H10	111.4	C9A—C14—H14B	129.5
C11—C10—H10	111.4	C13—C14—H14B	110.4
C11—C12—C13	111.2 (12)	H14A—C14—H14B	108.6
C11—C12—H12A	109.4	C8—N1—H3N	109 (2)
C13—C12—H12A	109.4	C8—N1—H2N	112 (3)
C11—C12—H12B	109.4	H3N—N1—H2N	105 (4)
C13—C12—H12B	109.4	C8—N1—H1N	104 (3)
H12A—C12—H12B	108.0	H3N—N1—H1N	116 (4)
C14—C13—C12	111.1 (10)	H2N—N1—H1N	111 (4)
C14—C13—H13A	109.4	C9—O1—C9A	31.2 (3)
C12—C13—H13A	109.4	C9—O1—P1	107.3 (4)
C14—C13—H13B	109.4	C9A—O1—P1	106.5 (4)
C12—C13—H13B	109.4	C10—O2—C10A	33.4 (4)
H13A—C13—H13B	108.0	C10—O2—P1	106.6 (4)
C10A—C9A—O1	102.8 (7)	C10A—O2—P1	107.4 (4)
C10A—C9A—C14	107.4 (7)	O4—P1—O3	115.7 (8)
O1—C9A—C14	110.5 (7)	O4—P1—O4A	20 (3)
C10A—C9A—H9A	111.9	O3—P1—O4A	115.4 (12)
O1—C9A—H9A	111.9	O4—P1—O2	104 (3)
C14—C9A—H9A	111.9	O3—P1—O2	110.38 (16)
C9A—C10A—C11	109.4 (8)	O4A—P1—O2	120.3 (14)
C9A—C10A—O2	96.3 (6)	O4—P1—O1	118 (3)
C11—C10A—O2	111.6 (6)	O3—P1—O1	109.49 (16)
C9A—C10A—H10A	112.8	O4A—P1—O1	101.8 (11)
C11—C10A—H10A	112.8	O2—P1—O1	96.79 (13)
			
C7—C2—C3—C4	0.1 (6)	C10—C9—C14—C13	63.4 (10)
C1—C2—C3—C4	−177.9 (4)	C12A—C13A—C14—C9	30.4 (14)
C2—C3—C4—C5	0.1 (6)	C12A—C13A—C14—C9A	61.0 (13)
C3—C4—C5—C6	−0.5 (5)	C12A—C13A—C14—C13	−48 (3)
C3—C4—C5—C8	176.4 (3)	C10A—C9A—C14—C9	49.1 (12)
C4—C5—C6—C7	0.8 (6)	O1—C9A—C14—C9	−62.3 (11)
C8—C5—C6—C7	−176.3 (3)	C10A—C9A—C14—C13A	−65.2 (11)
C5—C6—C7—C2	−0.7 (6)	O1—C9A—C14—C13A	−176.6 (8)
C3—C2—C7—C6	0.2 (6)	C10A—C9A—C14—C13	−36.9 (11)
C1—C2—C7—C6	178.2 (4)	O1—C9A—C14—C13	−148.2 (7)
C6—C5—C8—N1	−156.8 (3)	C12—C13—C14—C9	−55.5 (15)
C4—C5—C8—N1	26.3 (5)	C12—C13—C14—C13A	57 (3)
O1—C9—C10—O2	53.9 (7)	C12—C13—C14—C9A	−23.7 (16)
C14—C9—C10—O2	174.9 (5)	C14—C9—O1—C9A	−70.4 (12)
O1—C9—C10—C11	172.2 (5)	C10—C9—O1—C9A	47.9 (12)
C14—C9—C10—C11	−66.8 (9)	C14—C9—O1—P1	−163.7 (5)
C11—C12—C13—C14	54.8 (18)	C10—C9—O1—P1	−45.4 (7)
O1—C9A—C10A—C11	−169.5 (5)	C10A—C9A—O1—C9	−53.4 (12)
C14—C9A—C10A—C11	73.9 (9)	C14—C9A—O1—C9	60.9 (11)
O1—C9A—C10A—O2	−53.9 (7)	C10A—C9A—O1—P1	43.0 (7)
C14—C9A—C10A—O2	−170.5 (6)	C14—C9A—O1—P1	157.4 (5)
C11—C12A—C13A—C14	−61.2 (15)	C9—C10—O2—C10A	54.3 (10)
C9A—C10A—C11—C12	−42.3 (11)	C11—C10—O2—C10A	−61.0 (10)
O2—C10A—C11—C12	−147.6 (8)	C9—C10—O2—P1	−42.3 (7)
C9A—C10A—C11—C10	45.1 (9)	C11—C10—O2—P1	−157.6 (5)
O2—C10A—C11—C10	−60.2 (8)	C9A—C10A—O2—C10	−46.7 (10)
C9A—C10A—C11—C12A	−67.5 (10)	C11—C10A—O2—C10	67.1 (10)
O2—C10A—C11—C12A	−172.8 (8)	C9A—C10A—O2—P1	47.2 (7)
C13—C12—C11—C10A	−21.1 (16)	C11—C10A—O2—P1	161.0 (5)
C13—C12—C11—C10	−56.1 (15)	C10—O2—P1—O4	−108 (3)
C13—C12—C11—C12A	46 (3)	C10A—O2—P1—O4	−143 (3)
O2—C10—C11—C10A	67.5 (10)	C10—O2—P1—O3	127.1 (4)
C9—C10—C11—C10A	−44.6 (9)	C10A—O2—P1—O3	92.1 (5)
O2—C10—C11—C12	173.3 (6)	C10—O2—P1—O4A	−94.5 (13)
C9—C10—C11—C12	61.2 (10)	C10A—O2—P1—O4A	−129.5 (13)
O2—C10—C11—C12A	144.8 (7)	C10—O2—P1—O1	13.4 (4)
C9—C10—C11—C12A	32.7 (11)	C10A—O2—P1—O1	−21.6 (5)
C13A—C12A—C11—C10A	61.4 (15)	C9—O1—P1—O4	131 (3)
C13A—C12A—C11—C12	−59 (3)	C9A—O1—P1—O4	98 (3)
C13A—C12A—C11—C10	28.6 (16)	C9—O1—P1—O3	−93.8 (4)
O1—C9—C14—C13A	145.1 (7)	C9A—O1—P1—O3	−126.4 (4)
C10—C9—C14—C13A	35.1 (11)	C9—O1—P1—O4A	143.6 (15)
O1—C9—C14—C9A	68.6 (12)	C9A—O1—P1—O4A	111.0 (15)
C10—C9—C14—C9A	−41.4 (11)	C9—O1—P1—O2	20.7 (5)
O1—C9—C14—C13	173.4 (7)	C9A—O1—P1—O2	−11.9 (4)

### Hydrogen-bond geometry (Å, °)

**Table:**

*D*—H···*A*	*D*—H	H···*A*	*D*···*A*	*D*—H···*A*
N1—H3N···O3^i^	0.92 (4)	1.90 (4)	2.788 (5)	161 (3)
N1—H2N···O3^ii^	1.00 (6)	1.86 (6)	2.834 (5)	164 (4)
N1—H1N···O4^iii^	0.92 (5)	1.72 (6)	2.63 (2)	171 (5)

Symmetry codes: (i) −*x*+1, −*y*+1, −*z*+1; (ii) *x*, *y*+1, *z*; (iii) *x*+1, *y*+1, *z*.
